# Impact of Ageing on Pea Protein Volatile Compounds and Correlation with Odor

**DOI:** 10.3390/molecules27030852

**Published:** 2022-01-27

**Authors:** Estelle Fischer, Rémy Cachon, Nathalie Cayot

**Affiliations:** AgroSup Dijon, Université Bourgogne Franche-Comté, PAM UMR A 02.102, F-21000 Dijon, France; estelle.fischer@agrosupdijon.fr (E.F.); remy.cachon@agrosupdijon.fr (R.C.)

**Keywords:** pea protein, storage conditions, aroma, HS-SPME-GC-MS, ‘beany’ off-flavor

## Abstract

Vegetal proteins are of high interest for their many positive aspects, but their ‘beany’ off-flavor is still limiting the consumer’s acceptance. The aim of this work was to investigate the conservation of pea protein isolate (PPI) during time and especially the evolution of their organoleptic quality under two storage conditions. The evolution of the volatile compounds, the odor and the color of a PPI has been investigated during one year of storage. PPI was exposed to two treatments mimicking a lack of control of storage conditions: treatment A with light exposition at ambient temperature (A—Light 20 °C) and treatment B in the dark but with a higher temperature (B—Dark 30 °C). For each sampling time (0, 3, 6, 9, 12 months), the volatile compounds were determined using HS-SPME-GC-MS, the odor using direct sniffing, and the color using the measurement of L*, a*, b* parameters. Treatment A was the most deteriorating and led to a strong increase in the total volatile compounds amount, an odor deterioration, and a color change. Furthermore, a tentative correlation between instrumental data on volatile compounds and the perceived odor was proposed. By the representation of volatile compounds sorted by their sensory descriptor, it could be possible to predict an odor change with analytical data.

## 1. Introduction

The demand for plant-based proteins is increasing due to their numerous positive effects [1,2,3]. Many studies are dealing with the sensory aspects of these proteins [4,5], especially the ‘beany’ off-flavor, as it is limiting the consumer’s acceptance [6,7,8]. Numerous works are done on the off-flavor characterization, and on the impact of various processes on the profile in volatile compounds [9,10,11,12,13].

The typical ‘beany’ off-flavor, associated with ‘green’ and ‘earthy’ attributes, is a combination of different volatile compounds belonging to various chemical families like aldehydes, ketones, or alcohols [7,10]. These compounds are typically found in pulses or other legumes and are generated through different pathways, like enzymatic and non-enzymatic degradation of lipids, amino-acids and peptides [6,14]. Lipid oxidation starts early and rapidly after the harvest and during the first stages of the production of pea protein isolate (PPI) [6,10]. The ‘beany’ off-flavor is generated in early phases and can evolve during the storage.

**Scheme 1:**
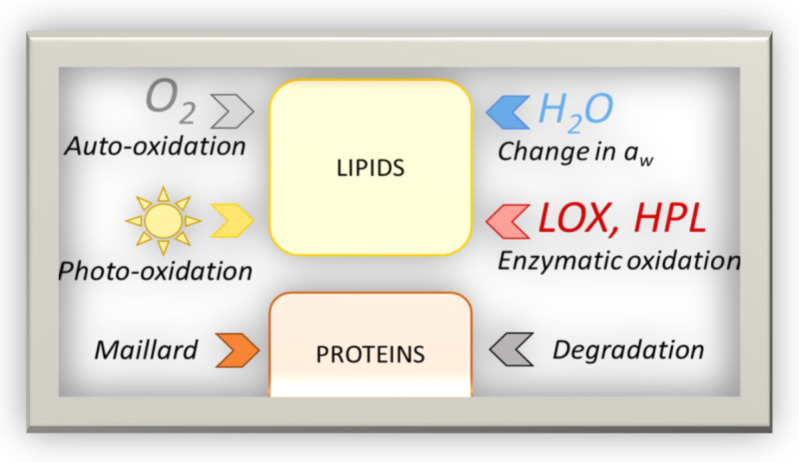
Reactions involved during the ageing [16,18,19,20].

Especially, this off-flavor can evolve quickly in PPI powder, becoming detrimental to the product. From the humidity/water activity (a_w_) relation to oxidation sensitivity, powders are highly sensible to lipid oxidation [15]. The lipid oxidation is very fast and can start since the harvest of the raw material. Lipids are rapidly converted into fatty acid hydroperoxydes (HPOD), leading to the formation of volatile and non-volatile compounds [16]. The primary products (HPOD) appear as early as seven days after the start of the cells breaking, and secondary products (volatile compounds) generally appear after the 15th day [17]. In this way, just after fabrication, PPI we used in the present study already contained all the volatile compounds resulting from lipid oxidation, originating from the harvest, the fabrication of PPI and the first months of storage. The first substrates, the lipids, generated the profile in volatile compounds of the product, by enzymatic and non-enzymatic oxidations. A_w_ changes during the storage could also impact the oxidation sensitivity. Scheme 1 presents the different phenomena affecting PPI.

Optimal storage conditions are required to limit the off-flavor worsening and the product deterioration. Some studies have already dealt with the evolution of volatile compounds (responsible for the ‘beany’ off-flavor) during the storage [21,22]. As an example, Schindler et al. (2011) analyzed the impact of transformation processes on the preservation of PPI under good storage conditions, and Azarnia et al. (2011) the impact of the storage temperature on different cultivars of raw peas. The present paper comes as a complementary study, by focusing on the impact of two important parameters of storage on a PPI: light and temperature.

This paper investigated the evolution of volatile compounds, of the odor, and of color of PPI during two storage treatments. The aim was to link the evolution of the volatile compounds with the odor deterioration and to observe the impact of storage parameters. The change of color of the PPI was also investigated as an external indicator of the conservation of the product. The storage conditions selection was guided towards abnormal storage conditions where environmental parameters were poorly controlled. Thus, treatment A was selected, with the product stored at 20 °C [22] and exposed to ambient light, mimicking a lack of control on the light exposition. Secondly, treatment B was selected, with the product stored at 30 °C in the dark, mimicking control over the light exposition but a lack of control over the temperature (increase in the storage temperature).

Firstly, the evolution of the volatile compounds, of the odor, and of color during the two storage treatments, were investigated. Then, hypotheses on the mechanisms involved in this evolution were proposed. Nine volatile compounds of interest were subsequently semi-quantified in the PPI. Hexanal, nonanal, 2-nonenal, 3-methylbutanal, 1-octen-3-ol, 3-octen-2-one, 2-pentylfuran, benzaldehyde, and 2,5-dimethylpyrazine were selected due to their reported involvement in the ‘beany’ off-flavor [7,10,23,24]. Finally, a way of predicting the odor using the evolution of the volatile compounds was considered to improve the use of instrumental data for early detection of the off-flavor evolution.

## 2. Results and Discussion

### 2.1. Color Evolution

The evolution of the color parameters (L*, a*, b*), during the two storage conditions and over twelve months was presented in Table 1. The treatment B (30 °C—Dark) had a small impact on the color. At six months, the sample became slightly darker (L* diminution) and, after three months, a little bit more red (a* increase) and less yellow (b* diminution). The storage in the dark at 30 °C was slightly affecting the sample color. The temperature increase enhanced Maillard reactions, leading to a color development and the sample browning [25]. On the other hand, treatment A (20 °C—Light) was leading to a drastic color change. From three to nine months, the sample became lighter (L* increase), less red (a* diminution); and from three to six months, less yellow (b* diminution). The sample color was strongly impacted by the exposition to light, as it can be seen with the ΔE* presented in the bottom of Table 1. ΔE* indicates the color difference between the color at the different treatment times and the original color at t0. The observed phenomenon could be explained by the carotenoids degradation caused by the light, leading to the product whitening [26]. The color measurement can then be a marker of an abnormal storage, where the product was exposed to light.

### 2.2. Odor Evolution

The evolution of the odor during the two storage treatments was presented in Table 2. The evaluation of the odor showed that treatment B (30 °C—Dark) did not really affect the odor. The product developed a ‘roasted’ attribute. This new attribute could be attributed to the Maillard reaction [27]. Especially, the ‘roasted’ attribute could arise from the lysine, threonine, and leucine involvement in the Maillard reaction [28]. These amino acids and particularly lysine and leucine were well present in pea protein, as presented in the Appendix A, Table A1. Treatment A (20 °C—Light) led to an odor deterioration as early as three months, with an increase in the ‘beany’ and ‘earthy’ attributes, and later on with the development of ‘rancid’ and ‘sulfurous’ off-notes. The increase of those attributes could be linked to lipid oxidation [16]. The ‘rancid’ attribute occurred from a strong increase of these reactions, leading to a quick deterioration of the product [29].

### 2.3. Volatile Compounds Evolution

#### 2.3.1. Total Chromatographic Area

**Figure 1:**
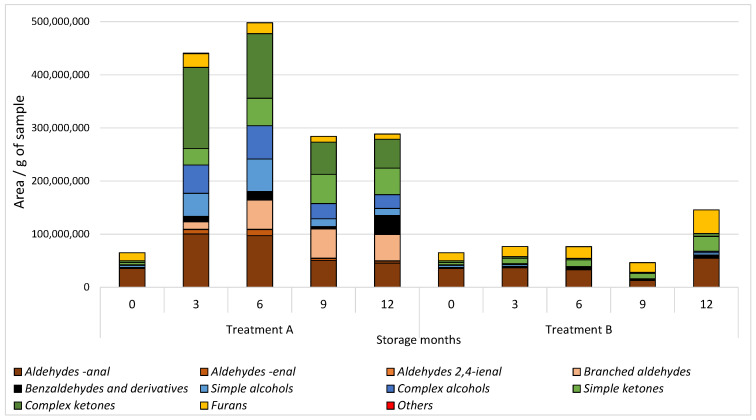
Profile of volatile compounds by chemical family during the ageing (*n* = 3). Treatment A: 20 °C—Light, Treatment B: 30 °C—Dark.

Figure 1 presented the evolution of the profile in volatile compounds in total chromatographic area per gram of sample, sorted by chemical families, during the two ageing treatments. The evolution of the full list of volatile compounds during the ageing can be found in Appendix A, Table A2, with the sorting of compounds into the different chemical families.

Treatment B (30 °C—Dark) had a slight impact on the volatile compounds, with mainly an increase at 12 months [13], correlated with a slight odor change. Lipid oxidation was not amplified. An increase in aldehydes and furans could however be observed, which could be attributed to the Maillard reactions amplified by the temperature. Indeed, Maillard reactions led to the formation of benzaldehyde (amino acids degradation from xylose and phenylalanine [18]) and furans [19]. Moreover, the protein degradation phenomenon can be observed, leading to the appearance of new compounds [20]. This phenomenon was slower than lipid oxidation, as it needed the amino acids and peptide release after proteolysis. Consequently, 3-methylbutanal, a compound resulting from the protein degradation [2], was detected at 12 months.

**Figure 2:**
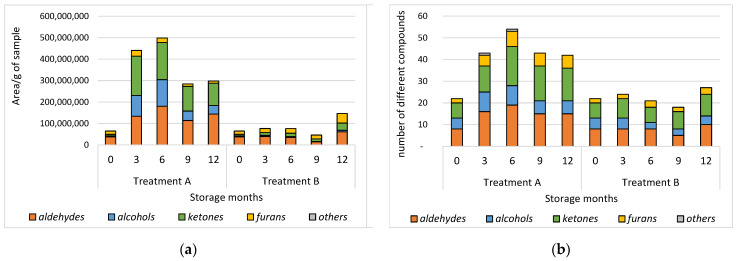
(**a**) Evolution of the total amount of volatile compounds during the ageing (*n* = 3); (**b**) Evolution of the number of different volatile compounds during the ageing (*n* = 3). Treatment A: 20 °C—Light, Treatment B: 30 °C—Dark.

Treatment A (20 °C—Light) had a strong impact on the volatile compounds. A high increase in the total of volatile compounds can be observed from zero to six months, followed by a small decrease at 9 months and an equilibrium at 12 months. This increase in the amount of volatile compounds was linked to the odor deterioration. An increase in the amount and diversity of aldehydes, alcohols and ketones could be observed, with the development of new compounds. In treatment A, the photo-oxidation due to light was added to the other phenomena, playing a crucial role in the formation of volatile compounds. From zero to three months, the highest increase in all types of volatile compounds, especially ketones and aldehydes, could be observed. This strong increase was mainly due to the formation of new compounds, derived from substrates still available. An increase in the furans diversity was also observed: new furans were formed, likely from the photo-oxidation of carotenoids [19]. The amount of volatile compounds increased, then slowed down between three and six months, and decreased at nine months. In order to explain this observation, the total amount of volatile compounds was compared to the number of different volatile compounds in Figure 2. According to these results, the lowering of the amount of volatile compounds at nine months was mainly due to the diminution of each volatile compound and did not result from the diminution of the diversity in compounds. As the literature generally focus on the early detection and the early stages of lipid oxidation [30], no precise phenomenon could be identified for this lowering. Volatile compounds can be followed to investigate ripening in fruits or food spoilage for example [31] with appearance of new targeted compounds. However, the volatile compounds fate and transformations over time are not well known. Some hypotheses can be suggested to explain the lowering in the amount of volatile compounds, like the transformation of the volatile compounds in other compounds, for instance non-volatile compounds. Other phenomena could also be involved. For example, reactions between the compounds themselves, reactions between compounds and the food matrix as it evolves during the storage, or a retention of the compounds by the packaging. Finally, between 9 and 12 months, for treatment A (20 °C—Light), the counts of volatile compounds were stable; the product reached an equilibrium.

In conclusion, within the range of temperatures studied, corresponding to usual storage conditions, light had a higher impact than temperature on the volatile compounds of the product.

#### 2.3.2. Semi-Quantification of Compounds of Interest

Nine interesting compounds involved in the ‘beany’ off-flavor were studied more in depth and were semi-quantified in the samples. These compounds were: hexanal, nonanal, 2-nonenal, 3-methylbutanal, benzaldehyde, 1-octen-3-ol, 3-octen-2-one, 2-pentylfuran, and 2,5-dimethylpyrazine. The evolution of these compounds during the two ageing conditions was presented in Table 3.

During treatment B (30 °C—Dark), as observed with the total chromatographic area, volatile compounds were slightly impacted. The amount of 3-octen-2-one, benzaldehyde and 2-pentylfuran increased at 3 or 6 months and the amount of 1-octen-3-ol, 3-methylbutanal and hexanal at 12 months. For all compounds, except nonanal, the highest increase for the amount of each compound could be observed at 12 months. The amount of nonanal was the only one to diminish during the ageing. 2-nonenal and 2,5-dimethylpyrazine were not detected during treatment B (30 °C—Dark). Treatment A (20 °C—Light) led to a higher increase in concentration in the studied volatile compounds. The amount of hexanal, nonanal, and 1-octen-3-ol increased at 3 and 6 months, followed by a diminution at 9 months, and a stabilization at 12 months. The amount of 3-octen-2-one followed the same evolution but the diminution started at six months. The amount of benzaldehyde strongly increased at 12 months and 2-nonenal appeared at 6 months. 2,5-dimethylpyrazine and 3-methylbutanal were not detected during treatment A (20 °C—Light).

This semi-quantification step allowed confirming the observations made previously with the total chromatographic area. For example, 3-methylbutanal was detected only at 12 months in treatment B (30 °C—Dark) and the amount of aldehydes strongly increased during the first months of treatment A (20 °C—Light). The amounts of volatile compounds found during treatment A (20 °C—Light) were very high. For example, the hexanal amount obtained at 3 months in treatment A was not obtained before 12 months using treatment B.

### 2.4. Correlation between Instrumental Data and Odor

**Figure 3:**
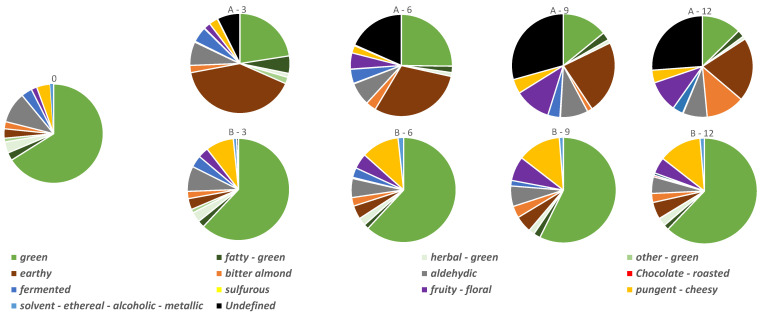
Profile in ratios of volatile compounds sorted by sensory descriptors during the two ageing conditions (*n* = 3). A given descriptor was represented as the ratio of the percentage of the cumulative amount of the volatile compounds responsible for the descriptor to the total amount of all volatile compounds. Treatment A: 20 °C—Light, Treatment B: 30 °C—Dark.

The odor evolution of the PPI seemed to be linked to the instrumental data of the volatile compounds. For example, the odor deterioration at three months in treatment A (20 °C—Light) matched with the increase of the amount of volatile compounds. The results used for Figure 1 were processed differently in order to investigate if instrumental data could predict the odor evolution. For all the volatile compounds identified in the product, the sensory descriptors were looked up [32] and the compounds were classified into different attribute families such as ‘green’ or ‘earthy’ as presented in Appendix A, Table A3. As the ‘beany’ off-flavor was investigated here, a focus was made on its different attributes, like the different types of ‘green’. A given descriptor was then represented as the ratio of the percentage of the cumulative amount of the different volatile compounds responsible for the descriptor, to the total amount of all volatile compounds. The evolution of these different sensory attributes during the two ageing conditions were presented in Figure 3.

During treatment B (30 °C—Dark), the profile of volatile compounds sorted by sensory descriptor was slightly evolving. Compounds responsible for the ‘pungent-cheesy’ attribute increased. During treatment A (20 °C—Light), compounds responsible for the ‘herbal-green’ attribute decreased and compounds responsible for ‘fatty-green’, ‘earthy’, ‘bitter almond’, and ‘fruity-floral’ attributes increased.

With this representation, it was possible to see a link between the instrumental data and the odor. At three months in treatment A (20 °C—Light), an ‘earthy’ off-flavor was perceived, as presented in Table 1, and this was linked to an increase in the proportion of volatile compounds responsible for ‘earthy’ notes presented in Figure 3. In the same way, for three and six months in treatment B (30 °C—Dark), little or no changes in the odor were smelled, and no big changes were seen in the ratios of volatile compounds. With the representation used in Figure 3 (volatile compounds sorted by sensory descriptors), changes in the ratios of the different descriptors were linked to the perception of new sensory attributes. Following these changes could help for the early detection of new off-flavors in a product.

However, there was a limit to these conclusions. This representation (Figure 3) was not sufficient to describe the global sensory profile of a product and this might not work with all attributes. For example, the increase in the proportion of compounds related to ‘fruity’ or ‘floral’ notes in treatment A (20 °C—Light) at 9 months was not perceived during the odor evaluation. To explain these phenomena, instrumental data needed to be checked together with information about the volatile compounds, like their perception threshold, their intensity and their interaction type with other types of compounds. Due to their very low perception threshold and high odor intensity [6,7], ‘beany’ and ‘rancid’ volatile compounds might conceal the sensory aspects of ‘fruity’ and ‘floral’ compounds. Moreover, with a ‘destructive’ sensory interaction between ‘beany’/’rancid’ and ‘fruity’/’floral’ compounds, one sensory aspect might completely erase the other one [33].

## 3. Materials and Methods

### 3.1. Sample

A spray-dried pea protein isolate (85% protein dry matter, composed mainly of globulins) was supplied by Roquette Frères S.A.

### 3.2. Ageing of the Samples

Approximately 20 g of the PPI were placed flat in plastic bags and exposed to the two storage treatments for 12 months: treatment A—20°C exposed to light, and treatment B—30°C in the dark. The PPI was sampled at 0, 3, 6, 9, and 12 months. At each sampling time, 2 g of sample was placed in a clear storage glass vial, and the different analyses were conducted directly after sampling.

### 3.3. Color Measurement

The color of the samples was determined with a Chromatometer CR-400 Konica Minolta. Three color parameters, L* (lightness), a* (redness) and b* (yellowness) were measured. The analysis was conducted directly on the samples in the plastic bags before sampling, with five measuring points flashed in triplicates. ΔE*, the color difference between the color at the different treatment times and the original color at t0, was calculated as follows with Equation (1).

(1) $$\begin{array}{c} \Delta E{}^{*}\;=\sqrt{{\left({L{}^{*}}_{2}-{L{}^{*}}_{1}\right)}^{2}+{\left({a{}^{*}}_{2}-{a{}^{*}}_{1}\right)}^{2}+\left({b{}^{*}}_{2}-{b{}^{*}}_{1}\right)²}\\ {L{}^{*}}_{1},{\;a{}^{*}}_{1},{\;b{}^{*}}_{1}:\mathrm{original}\;\mathrm{color}\;\mathrm{parameters}\;\mathrm{at}\;t0\\ {L{}^{*}}_{2},{\;a{}^{*}}_{2},{\;b{}^{*}}_{2}\;:\mathrm{color}\;\mathrm{parameters}\;\mathrm{during}\;\mathrm{the}\;\mathrm{treatments} \end{array}$$

### 3.4. Odor Determination

The odor was determined by direct sniffing of the product in the storage glass vial. At least 24 h after sampling, the products were smelled into the glass vial by three experts, directly at the opening of the vial. The odor character and intensity were descripted and the enounced sensory descriptors of the sample were recorded [34]. Especially, the products were evaluated toward the ‘beany’, ‘earthy’, and ‘rancid’ attributes. The ‘beany’ attribute was separated in three categories: light ‘beany’ when the attribute was slightly present; ‘beany’ when the attribute was well present and ‘beany’ + when the attribute was predominant or had a high intensity. The adjective ‘strong’ was used to indicate that the odor of the product was globally more intense.

### 3.5. Volatile Compounds Analysis

The volatile compounds extraction was done using headspace solid micro-extraction and the analysis using gas chromatography coupled with mass spectrophotometry (HS-SPME-GC-MS). For each treatment at each sampling time, volatile compounds analysis was run in triplicates, using a previously optimized method for PPI [35].

From the 2 g aliquot, a 0.2 g PPI sample was weighted directly in a new clear 20 mL extraction vial (VA201) capped with septum caps (18 mm caps, 8 mm PTFE/silicon septum, SACA001), all purchased from JASCO, France. Distilled water was added to obtain a 2 mL suspension at 10% (*w*/*v*) and a liquid/gas ratio of 2/18 (*v*/*v*). A SPME device containing a 1 cm fused-silica fiber coated with a 50/30 μm thickness of DVB/CAR/PDMS (divinylbenzene/carboxen/polydimethylsiloxane) was used for HS-SPME extraction. This fiber was selected to ensure the best extraction of a diversity of volatile compounds [9,10,36,37,38]. The fiber (24 Ga 50/30 μm, for manual holder, 3 pK, 57328-U) was purchased from Sigma and used with a manual fiber holder. The extractions were carried out in an electro thermal magnetic stirrer with a water bath (MS-H-Pro+, DLAB) to ensure a homogeneous temperature and constant agitation for the sample and headspace. The fiber was conditioned before analysis by heating it in the gas chromatograph injection port at 270 °C for 30 min, according to the manufacturer’s specifications. Equilibrium step and extraction step were conducted both at 40 °C with agitation at 350 rpm in the dark. The equilibrium time was 30 min and the extraction time, exposure of the fiber in the headspace of the vial was 60 min [9,10,13,36,37,38,39,40,41,42,43].

An HP 6890 Series Gas Chromatograph (Hewlett-Packard, Palo Alto, CA, USA) equipped with an HP 5973 Mass Selective Detector (Agilent Technologies, Palo Alto, CA, USA) (Quadrupole) was used with a DB-WAX column (30 m × 0.32 mm × 0.25μm, 123-7032, Agilent, J&W Scientific, Santa Clara, United States) to analyze the compounds of interest [10,13,21,23]. The SPME fiber was desorbed and maintained in the injection port at 250 °C for 5 min. The sample was injected in split mode, with a purge flow of 140 mL/min at 0 min to generate sharp, well-separated peaks on the chromatograph. Helium was used as a carrier gas at 1.4 mL/min with a linear velocity of 43 cm/s. The programmed temperature, selected from preliminary trials, was isothermal at 40 °C for 3 min, raised to 100 °C at a rate of 3 °C/min, and then raised to 230 °C at a rate of 5 °C/min and held for 10 min. The total run time was 59 min [13,21]. The ionization source and transfer line temperatures were set respectively at 230 °C and 190 °C.

The mass spectra were obtained using a mass selective detector with an electron impact voltage of 70 eV in full scan mode over the range *m*/*z* 29 to 400. Compounds were identified by comparing their mass spectra with NIST 08 (National Institute of Standards and Technology), Wiley, and INRA libraries, with a low integration limit of 50,000 in peak area, allowing the best peak identification.

### 3.6. Semi-Quantification

Nine compounds of interest were semi-quantified in the PPI, due to their involvement in the ‘beany’ off-flavor [7,10,23,24]. The following standards were purchased from Sigma-Aldrich: hexanal (98% purity, CAS 66-25-1), nonanal (>98%, CAS 124-19-6), trans-2-nonenal (97%, CAS 18829-56-6), 3-methylbutanal (97%, CAS 590-86-3), 1-octen-3-ol (98%, CAS 3391-86-4), 3-octen-2-one (98%, CAS 1669-44-9), 2-pentylfuran (98%, CAS 3777-69-3), benzaldehyde (99%, CAS 100-52-7), and 2,5-dimethylpyrazine (98%, CAS 123-32-0). An external calibration method, previously optimized for PPI [35], was used. The calibration curves of each of the nine compounds were obtained for concentrations ranging from 0.001 to 2.5 ppm, in distilled water. The amount of each compound in the sample was calculated as in the following example with hexanal. Semi-quantification steps were as following, with a and b from the calibration curve of hexanal (a = slope, b = intercept of the regression):

Area Hexanal = 11585856 A.U.(2)

[Hexanal]_in the assay_ (µg/mL) = (Area hexanal − b)/a = (11585856−157037)/1 × 10^7^ = 1.14 µg/mL (3)

[Hexanal]_in the sample_ (µg/g) = ([Hexanal]_in the assay_ (µg/mL) × V_solution_ (mL))/m_sample_ (g) = (1.14 × 2)/0.2078 = 11.0 µg/g(4)

m_[Hexanal]in the sample_ (*n* = 3) = 10.7 ± 0.3 µg of hexanal/g of PPI (5)

### 3.7. Data Treatment

The statistical treatment of color data and semi-quantification data was performed using the software Minitab 18 (Minitab, LLC., State College, Pennsylvania, United States). One-way analysis of variance (ANOVA) was applied for all the results after validating the feasibility of the test using variance analysis. Significance was established at *p* < 0.05. ANOVA showing significant differences lead to the use of Tukey’s multiple comparison test to group the samples.

Volatile compounds were first sorted by chemical family to obtain Figure 1. To obtain Figure 3, volatile compounds were sorted by sensory descriptor [32] as presented in Appendix A, Table A3. A given descriptor was then represented as the ratio of the percentage of the cumulative amount of the different volatile compounds responsible for the descriptor, to the total amount of all volatile compounds.

## 4. Conclusions

To conclude, the storage conditions had a strong impact on the volatile compounds, on the odor, and on the color of the studied PPI. This work highlighted the importance of light exposition and the crucial role of the first three months of storage. Indeed, light exposition was detrimental to the color and the odor of PPI, and a high increase in the total amount of volatile compounds was observed.

Conservation of PPI or other pulses proteins must be achieved in the dark of with light impermeable packaging to prevent the light exposition. This work also highlighted the fate of volatile compounds in long-term ageing: past six months, the amount of volatile compounds started to decrease to reach an equilibrium. In association with the volatile compounds, the evolution of the color could be a good indicator to follow, to detect an abnormal evolution of the product. The amino acid, fatty acid, and hydrolysis state of the product are important factors that may also affect the evolution of the product during the storage.

When representing analytical data using sensory descriptors, it was possible to predict how compounds responsible for different attributes evolved. This could be used for early detection of an odor change, by detecting a change in the ratios of compounds responsible for the different studied attributes.

With the increasing use of vegetable proteins in food products, their storage conditions and conservation over time must be carefully studied and established, as small variations could potentially lead to detrimental sensory impacts. Environmental parameters should be controlled to optimize conservation, especially with intermediate products such as PPI that are highly sensible to oxidation. These recommendations could however be advised also for the storage of raw materials such as raw peas, or final products containing PPI.

## Figures and Tables

**Table 1:** Color evolution during the ageing (*n* = 5 points in triplicates).

Months	0	3	6	9	12
Treatment A—20 °C Light					
L*	82.9 ± 0.2 ^d^	87.0 ± 0.3 ^c^	88.2 ± 0.5 ^b^	88.9 ± 0.2 ^a^	88.8 ± 0.2 ^a,b^
a*	3.14 ± 0.06 ^a^	2.3 ± 0.2 ^b^	1.9 ± 0.3 ^c^	1.37 ± 0.03 ^d^	1.50 ± 0.05 ^d^
b*	20.84 ± 0.08 ^a^	11.1 ± 0.4 ^b^	10.6 ± 0.5 ^c^	10.4 ± 0.1 ^c^	10.7 ± 0.2 ^bc^
Treatment B—309 °C Dark					
L*	83.1 ± 0.2 ^a^	82.7 ± 0.3 ^a,b^	82.1 ± 0.1 ^c^	82.4 ± 0.2 ^b,c^	82.5 ± 0.3 ^b,c^
a*	3.21 ± 0.06 ^c^	3.39 ± 0.05 ^b^	3.62 ± 0.06 ^a^	3.66 ± 0.05 ^a^	3.62 ± 0.06 ^a^
b*	21.21 ± 0.08 ^a^	20.6 ± 0.2 ^b^	20.8 ± 0.2 ^b^	20.4 ± 0.2 ^b^	18.8 ± 0.3 ^c^
ΔE*					
Treatment A		10.6	11.6	12.2	11.8
Treatment B		0.8	1.2	1.2	2.5

^a–d^: Statistical groups for each parameter on each sample, during time. Different letters mean significant difference at the 0.05 level.

**Table 2:** Odor evolution during the ageing (evaluation by three experts).

	Treatment A (20 °C—Light)	Treatment B (30 °C—Dark)
0	Light ‘beany’	Light ‘beany’
3	Strong, ‘beany’, sharp, earthy	Light ‘beany’, roasted
6	Strong, ‘beany’ +, sharp, earthy	Light ‘beany’, roasted
9	‘Beany’ +, sharp, earthy, rancid	Light ‘beany’, roasted, earthy
12	‘Beany’+, sharp, earthy, rancid	‘Beany’, earthy

**Table 3:** Evolution of the nine compounds of interest during the ageing, in µg of compound/g of sample (*n* = 3).

Months	0	3	6	9	12
**Treatment A (20 °C—Light)**					
Hexanal	5.1 ± 0.2 ^c^	10.7 ± 0.3 ^a^	10.1 ± 0.1 ^b^	4.1 ± 0.2 ^d^	3.7 ± 0.1 ^d^
Nonanal	1.01 ± 0.08 ^c^	3.1 ± 0.3 ^a,b^	3.8 ± 0.5 ^a^	2.5 ± 0.4 ^b^	2.3 ± 0.5 ^b^
2-nonenal	n.d.	n.d	0.72	0.66 ± 0.07	0.58 ± 0.06
3-methylbutanal	n.d.	n.d.	n.d.	n.d.	n.d.
1-octen-3-ol	0.07 ± 0.01 ^d^	4.64 ± 0.07 ^b^	5.0 ± 0.1 ^a^	2.3 ± 0.1 ^c^	2.04 ± 0.06 ^c^
3-octen-2-one	0.08 ± 0.05 ^d^	24.5 ± 0.5 ^a^	18.1 ± 0.6 ^b^	7.1 ± 0.2 ^c^	6.6 ± 0.3 ^c^
2-pentylfuran	8.1 ± 0.8 ^a^	5.2 ± 0.4 ^b^	5.6 ± 0.8 ^b^	2.05 ± 0.07 ^c^	1.9 ± 0.1 ^c^
Benzaldehyde	0.41 ± 0.03 ^b^	2.9 ± 0.2 ^b^	5 ± 1 ^b^	3.80	10 ± 4 ^a^
2,5-dimethylpyrazine	n.d.	n.d.	n.d.	n.d.	n.d.
**Treatment B (30 °C—Dark)**					
Hexanal	5.1 ± 0.2 ^b^	5.5 ± 0.3 ^b^	5.1 ± 0.3 ^b^	1.68 ± 0.06 ^c^	9.3 ± 0.8 ^a^
Nonanal	1.01 ± 0.08 ^a^	0.84 ± 0.08 ^a^	0.56 ± 0.01 ^b^	0.31 ± 0.03 ^c^	0.9 ± 0.1 ^a^
2-nonenal	n.d.	n.d.	n.d.	n.d.	n.d.
3-methylbutanal	n.d.	n.d.	n.d.	n.d.	< 0.05
1-octen-3-ol	0.07 ± 0.01 ^b,c^	0.10 ± 0.01 ^b^	0.088 ± 0.004 ^b^	0.05 ± 0.01 ^c^	0.24 ± 0.02 ^a^
3-octen-2-one	0.08	0.08 ± 0.02 ^c^	0.20 ± 0.01 ^b^	0.13 ± 0.01 ^c^	0.53 ± 0.03 ^a^
2-pentylfuran	8.1 ± 0.8 ^c^	10 ± 1 ^bc^	11.1 ± 0.7 ^b^	9.5 ± 0.5 ^b,c^	22 ± 1 ^a^
Benzaldehyde	0.41 ± 0.03 ^c^	0.48 ± 0.03 ^b,c^	0.55 ± 0.02 ^b^	0.46 ± 0.03 ^b,c^	1.12 ± 0.06 ^a^
2,5-dimethylpyrazine	n.d.	n.d.	n.d.	n.d.	n.d.

^a–d^: Statistical groups for each compound in each sample during time; when no standard deviation was available, the compound was retrieved only in one of the three repetitions; n.d. = not detected; <0.05 = not quantified, below the quantification limit of the compound.

## Data Availability

Not applicable.

## Sample Availability

Samples are not available from the authors.

## Appendix A. Complementary Data

**Table A1:** Amino-acid composition of pea protein, g of amino acid/100 g of protein [44].

Amino Acids	Iso	Leu	Lys	Met	Phe	Thr	Trp	Val	Arg	His	Ala	Asp	Cys	Glu	Gly	Pro	Ser	Tyr
Pea	3.3	6.6	6.8	1.0	4.2	3.6	0.9	3.9	6.8	2.5	4.3	10.7	1.6	16.9	4.3	3.4	4.8	3.1

**Table A2:** Evolution of the volatile compounds during the two treatments, sorted by chemical families, in area/g of sample (*n* = 3).

						Treatment A (20 °C—Light)				Treatment B (30 °C—Dark)			
						Months of Ageing							
Compounds			CAS	rt	0	3	6	9	12	3	6	9	12
Aldehydes	“-anal”	Propanal	000123-38-6	1.59	-	552,702	-	-	-	-	-	-	-
		Butanal	000123-72-8	2.03	-	1,115,111	726,721	467,966	425,129	-	253,670	-	387,711
		Pentanal	000110-62-3	3.14	1,805,405	10,599,476	8,885,899	4,156,205	3,766,730	2,387,833	1,895,765	686,938	-
		Hexanal	000066-25-1	5.30	26,205,328	54,239,367	51,391,065	21,093,759	19,104,638	28,292,761	26,191,857	9,162,214	47,077,876
		Heptanal	000111-71-7	8.52	754,598	-	-	-	-	-	-	-	-
		Octanal	000124-13-0	12.73	1,121,496	17,757,367	16,909,904	11,651,259	10,524,072	1,473,869	1,281,903	954,109	2,678,665
		Nonanal	000124-19-6	17.03	5,465,053	16,049,897	19,359,518	13,001,001	11,876,720	4,624,696	3,207,973	1,963,300	4,751,584
	“-enal”	2-Hexenal	000505-57-7	9.56	345,986	-	1,043,789	589,469	526,462	252,575	283,100	-	360,970
		2-Heptenal, (*Z*)-	057266-86-1	13.99	357,735	5,405,526	5,979,637	2,585,616	2,352,529	357,216	265,512	-	474,315
		2-Octenal, (*E*)-	002548-87-0	18.30	-	2,667,469	3,688,839	-	-	248,380	-	-	364,173
		2-Nonenal, (*E*)-	018829-56-6	22.48	-	-	517,949	771,976	673,686	-	-	-	-
	“2,4-ienal”	2,4-Heptadienal, (*E*,*E*)-	004313-03-5	20.80	-	108,131	-	-	-	-	-	-	-
		2,4-Octadienal, (*E*,*E*)-	030361-28-5	6.69	-	-	206,327	-	-	-	-	-	-
		2,4-Nonadienal, (*E*,*E*)-	005910-87-2	27.95	-	695,412	722,214	583,815	528,861	-	-	-	-
	branched aldehydes	Butanal, 3-methyl-	000590-86-3	2.37	-	-	-	-	-	-	-	-	353,164
		2-Butenal, 2-ethyl-	019780-25-7	7.17	-	499,000	665,077	-	-	-	-	-	-
		2-Butenal, 2-methyl-, (*E*)-	000497-03-0	5.51	-	806,907	1,027,432	-	-	-	-	-	-
		2-Pentenal, 2-ethyl-	003491-57-4	10.88	-	-	1,473,437	569,633	518,283	-	-	-	-
		2-Pentenal, 2-methyl-	000623-36-9	7.40	-	-	-	-	-	-	-	-	82,402
		Hexenal, 2-ethyl-	026266-68-2	13.10	-	-	1,026,486	1,027,798	896,314	-	-	-	-
		2-Heptenal, 2-methyl-	030567-26-1	15.19	-	4,063,506	7,303,976	4,141,841	3,754,692	-	-	-	-
		2-Heptenal, 2-propyl-	034880-43-8	20.30	-	2,165,030	10,928,934	10,906,205	9,880,515	-	-	-	-
		2-Octenal, 2-butyl-	013019-16-4	27.21	-	6,537,644	32,205,624	38,025,304	34,502,265	-	-	-	-
	B	Benzaldehyde	000100-52-7	21.76	1,511,999	10,335,375	16,256,676	4,471,740	35,544,260	1,771,439	1,993,722	1,683,167	3,989,612
Alcohols	Simple alcohols	1-Butanol	000071-36-3	7.61	-	319,454	1,263,785	-					
		1-Pentanol	000071-41-0	11.68	502,134	11,367,530	12,396,492	6,571,583	5,965,497	523,409	429,986	88,978	1,010,269
		1-Hexanol	000111-27-3	10.07	2,364,526	2,994,313	2,661,025	1,143,420	1,032,407	2,359,375	1,856,811	833,988	3,109,508
		1-Heptanol	000111-70-6	20.02	248,472	7,115,800	14,254,574	2,968,518	2,720,978	290,207	-	-	376,430
		1-Octanol	000111-87-5	24.04	466,763	21,472,341	30,667,441	4,277,326	3,878,804	443,403	-	-	-
	Complex alcohols	1-Penten-3-ol	000616-25-1	8.09	-	355,276	997,392	-	-	-	-	-	-
		1-Octen-3-ol	003391-86-4	19.86	1,031,787	46,659,774	50,288,891	22,859,528	20,698,276	1,334,531	1,175,312	743,572	2,670,922
		2-Octen-1-ol, (*E*)-	018409-17-1	25.80	-	544,347	9,698,048	5,796,319	5,256,259	-	-	-	-
		1-Nonen-4-ol	035192-73-5	26.38	-	-	1,674,594	-	-	-	-	-	-
		2-Nonen-1-ol, (*E*)-	031502-14-4	25.89	-	5,807,586	-	-	-	-	-	-	-
Ketones	Simple ketones	Acetone	000067-64-1	1.69	-	-	191,413	-	-	-	-	-	-
		2-Butanone	000078-93-3	2.21	-	-	400,901	-	-	-	-	-	178,098
		2-Heptanone	000110-43-0	8.47	2,914,251	12,727,931	11,834,021	12,835,121	11,630,756	6,778,697	8,889,881	6,164,234	19,027,798
		2-Octanone	000111-13-7	12.60	363,899	4,698,052	7,159,877	4,813,146	4,359,318	645,326	758,827	705,801	1,988,617
		3-Octanone	000106-68-3	11.30	-	2,616,637	3,338,368	2,040,859	1,853,790	86,399	-	-	553,777
		2-Nonanone	000821-55-6	16.87	715,439	3,902,976	10,344,382	10,462,661	9,479,837	1,590,570	2,187,542	2,034,953	4,795,261
		3-Nonanone	000925-78-0	15.53	-	417,012	740,060	-	-	-	-	-	-
		4-Nonanone	004485-09-0	14.23	-	-	2,813,609	1,619,983	1,472,690	-	-	-	-
		2-Decanone	000693-54-9	21.10	476,536	2,763,256	7,534,404	9,596,606	8,698,039	968,225	1,038,001	1,254,108	1,629,600
		5-Decanone	000820-29-1	18.42	-	1,536,282	994,339	5,282,123	4,786,286	435,517	-	-	-
		2-Undecanone	000112-12-9	24.94	-	-	335,632	469,896	425,404	-	-	-	-
		6-Undecanone	000927-49-1	22.45	-	2,281,893	6,017,331	7,803,108	7,086,946				
	Complex ketones	3-Hepten-2-one, 5-methyl-	005090-16-4	14.33	-	-	18,668,068	9,738,639	8,833,310	-	-	-	-
		5-hepten-2-one, 6-methyl-	000110-93-0	14.58	-	-	-	-	-	-	301,548	181,797	385,650
		3-Cyclohepten-1-one	001121-64-8	2.81	-	-	1,304,173	-	-	-	-	-	-
		1-Octen-3-one	004312-99-6	13.24	-	2,143,093	1,628,850	685,187	624,964	-	-	-	-
		3-Octen-2-one	001669-44-9	17.63	609,220	124,925,658	90,943,451	36,567,885	32,817,049	686,237	1,276,001	932,929	2,900,763
		2,3-Octanedione	000585-25-1	14.44	796,320	9,271,454	-	-	-	889,117	-	-	-
		3,5-Octadien-2-one	038284-27-4	23.86	1,695,929	16,505,439	6,034,706	3,770,372	3,375,879	1,439,512	1,148,274	977,159	1,988,993
		3-Nonen-2-one	014309-57-0	21.59	-	-	2,989,705	7,195,393	6,526,713	-	-	-	-
		3-Decen-2-one	010519-33-2	25.46	-	-	-	2,649,693	2,400,948	-	-	-	-
Furans		Furan, 2-ethyl-	003208-16-0	2.73	11 604	-	-	-	-	823,078	1,328,127	578,699	1,793,100
		Furan, 2-ethyl-5-methyl-	001703-52-2	5.71	-	-	526,982	-	-	-	-	-	-
		Furan, 2-n-butyl-	004466-24-4	6.43	-	-	-	-	-	-	75,227	-	440,015
		Furan, 2-pentyl-	003777-69-3	10.34	14,612,684	8,692,818	9,484,780	2,299,038	2,088,023	17,878,803	20,570,994	17,427,094	41,966,438
		Furan, 2,3-dihydro-4-(1-methylpropyl)-, (*S*)-	034379-54-9	12.06	-	13,723,850	4,262,423	1,740,556	1,579,739	-	-	-	-
		(-)-(*R*)-5-Pentyl-2(5*H*)-furanone	091510-97-3	30.87	-	1,667,313	2,343,321	2,579,914	2,338,950	-	-	-	-
		2(3*H*)-Furanone, dihydro-5-pentyl-	000104-61-0	35.49	-	319,843	1,026,142	1,420,970	1,286,704	-	-	-	-
		2(3*H*)-Furanone, 5-butyldihydro-	000104-50-7	33.06	-	-	210,886	357,530	324,362	-	-	-	-
		5-pentyl-5(*H*)-furan-2-one	021963-26-8	36.44	-	1,646,695	2,381,544	2,545,614	2,304,622	-	-	-	-
S		Disulfide, dimethyl	000624-92-0	4.96	-	571,177	308,345	-	-	-	-	-	-

Legend: rt = retention time in minutes, S = sulfides, B = benzaldehyde.

**Table A3:** Sorting of volatile compounds by sensory descriptors.

Descriptor	Compounds	Descriptor	Compounds
Green	Hexanal	Sulfurous	Disulfide, dimethyl
	Heptanal	Fruity-floral	2-Nonanone
	2-Hexenal		2-Decanone
	2-Heptenal, (*E*)-		5-Hepten-2-one, 6-methyl-
	2,4-Heptadienal, (*E,E*)-		2-n-Butyl furan
	2,4-Nonadienal, (*E,E*)-		3-Nonanone
	1-Heptanol		(-)-(*R*)-5-Pentyl-2(5*H*)-furanone
	2-Octen-1-ol, (*E*)-		2(3*H*)-Furanone, dihydro-5-pentyl-
	Furan, 2-pentyl-		3-Nonen-2-one
	1-Octanol		2-Undecanone
	1-Penten-3-ol		2(3*H*)-Furanone, 5-butyldihydro-
	2-Butenal, 2-methyl-, (*E*)-	Pungent-cheesy	2-Heptanone
	2,4-Octadienal, (*E,E*)-	Solvent-ethereal-alcoholic-metallic	Acetone
Fatty-green	2-Octenal, (*E*)-		2-Butanone
	2-Nonenal, (*E*)- et (*Z*)-		Furan, 2-ethyl-
	2-Nonen-1-ol, (*E*)-		Propanal
	3,5-Octadien-2-one, (*E,E*)-		Furan, 2-ethyl-5-methyl-
	3-Decen-2-one	Undefined	2-Octenal, 2-butyl-
Herbal-green	1-Hexanol		3-Hepten-2-one, 5-methyl-
	3-Octanone		2-Butenal, 2-ethyl-
Other-green	2,3-Octanedione		2-Heptenal, 2-methyl-
	2-pentenal, 2-methyl		5-Decanone
Earthy	1-Octen-3-ol		6-Undecanone
	2-Octanone		Furan, 2,3-dihydro-4-(1-methylpropyl)-, (*S*)-
	1-Octen-3-one		5-pentyl-5(*H*)-furan-2-one
	3-Octen-2-one, (*E*)-		2-Heptenal, 2-propyl-
Bitter almond	Benzaldehyde		2-pentenal, 2-ethyl
Aldehydic	Octanal		Hexenal, 2-ethyl-
	Nonanal		1-Nonen-4-ol
Chocolate-roasted	Butanal, 3-methyl-		3-Cyclohepten-1-one
	Butanal		4-Nonanone
Fermented	Pentanal		
	1-Pentanol		
	1-Butanol		
